# Abstract of Meteorological Observations for February, 1855, Made at Philadelphia, Pa.

**Published:** 1855-04

**Authors:** James A. Kirkpatrick


*Abstract of Meteorological Observations for February,* 1855, *made at Philadelphia, Pa. Latitude* 39° 57′ 28″ *N., Longitude* 75° 10′ 40″ *W. from Greenwich.* By PROF. JAMES A. KIRKPATRICK.

| 1855. Feb. | Barometer. Daily Mean | Barometer. Mean Daily Range. | Thermom. Daily Mean | Thermom. Mean Daily Range | Dew Point 2 P. M. | Rel. Humid. 2 P. M. | Rain and melted Snow. | Prevailing Winds. | Remarks. |
|---|---|---|---|---|---|---|---|---|---|
| | Inches. | Inches. | Deg. | Deg. | Deg. | Hunds. | Inch. | Points. | |
| 1 | 29.850 | .172 | 27.7 | 2.2 | 28.7 | 0.89 | | NW. | M. and aft. cloudy; ev. clear. |
| 2 | 29.773 | .077 | 30.3 | 3.3 | 28.0 | .69 | | SW. | Cloudy. |
| 3 | 29.734 | .096 | 24.0 | 7.7 | 13.0 | .30 | | W. | Clear. |
| 4 | 29.795 | .098 | 20.0 | 7.3 | 12.7 | .45 | | W.NW. | M. and aft. clear; ev. cloudy. |
| 5 | 29.586 | .210 | 20.5 | 3.8 | 13.7 | .47 | 0.031 | NW. | M. rain; aft. and ev. clear. |
| 6 | 29.897 | .311 | 4.5 | 16.0 | 2.0 | .28 | | NW. | M. and aft. clear; ev. cloudy. |
| 7 | 29.967 | .216 | 5.7 | 6.8 | 3.0 | 1.00 | } | NE. | Snow. *Therm. lowest* 1°. |
| 8 | 29.585 | .382 | 22.5 | 16.8 | 24.3 | .93 | } 0.690 | N. | Snow. *Bar. lowest* 29.535. |
| 9 | 29.676 | .118 | 25.3 | 4.2 | 26.8 | .93 | } | N.NW. | *Snow,* about 5 inches. |
| 10 | 29.992 | .316 | 17.7 | 7.7 | 16.3 | .71 | | NW. | Clear. |
| 11 | 29.930 | .062 | 21.8 | 4.5 | 22.3 | .76 | | W.SW. | Cloudy. |
| 12 | 30.117 | .187 | 26.7 | 4.8 | 25.2 | .73 | | (Var.) | M. clear; aft. and ev. cloudy. |
| 13 | 30.086 | .074 | 35.0 | 8.3 | 33.7 | .67 | | NE. | Cloudy. |
| 14 | 29.733 | .352 | 43.3 | 8.3 | 45.3 | .96 | 1.748 | NE. | *Rain* all day. *Therm. highest* 46½°. |
| 15 | 29.577 | .156 | 39.8 | 3.5 | 39.7 | .91 | 0.011 | W.NW. | Cloudy; morning drizzling. |
| 16 | 29.694 | .117 | 37.7 | 2.2 | 28.3 | .56 | | W. | Cloudy. |
| 17 | 29.797 | .103 | 33.7 | 4.0 | 29.0 | .70 | | W. | M. and aft. cloudy; ev. clear. |
| 18 | 29.796 | .010 | 36.7 | 3.0 | 28.3 | .56 | | W.NW | Clear. |
| 19 | 29.790 | .026 | 35.0 | 1.7 | 26.3 | .54 | | NW. | M. cloudy; aft. and ev. clear. |
| 20 | 29.871 | .088 | 33.2 | 1.8 | 26.7 | .60 | | NW. | Clear. |
| 21 | 30.062 | .191 | 34.0 | 1.8 | 27.3 | .55 | | NW. | Clear. |
| 22 | 29.937 | .129 | 39.0 | 5.0 | 27.3 | .40 | | NW. | M. clear; aft. and ev. cloudy. |
| 23 | 29.829 | .114 | 30.0 | 15.0 | 26.7 | .89 | | N. | Cloudy. |
| 24 | 29.963 | .130 | 19.7 | 11.0 | 13.7 | .47 | | W. | M. and aft. clear; ev. cloudy. |
| 25 | 29.832 | .131 | 21.0 | 4.0 | 15.0 | .34 | | W. | Clear. |
| 26 | 29.686 | .146 | 17.7 | 3.3 | 11.7 | .43 | | W. | M. clear; aft. and ev. cloudy. |
| 27 | 29.848 | .162 | 20.7 | 3.0 | 18.0 | .64 | | NW. | M. and aft. cloudy; ev. clear. |
| 28 | 30.105 | .287 | 23.5 | 2.8 | 17.3 | .45 | | NW. | Clear. *Bar. highest* 30.199. |
| Means for Feb. 1855 | 29.840 | .159 | 26.7 | 5.8 | 22.4 | .71 | 2.480 | N. 50° W., 83–100. | |
| 4 yrs | 29.901 | | 32.3 | | 30.4 | | 3.458 | N. 62° W. 54–100. | |

The Monthly Range of the Barometer was 0.664 inch., and of the Thermometer 47½°

---

POSTSCRIPT.—We are requested to announce that Dr. Brown-Séquard has resigned the chair of the Institutes of Medicine, &c., in the Medical College of Virginia at Richmond.—ED.

